# Crucial transcripts predict response to initial immunoglobulin treatment in acute Kawasaki disease

**DOI:** 10.1038/s41598-020-75039-z

**Published:** 2020-10-20

**Authors:** Zhimin Geng, Jingjing Liu, Jian Hu, Ying Wang, Yijing Tao, Fenglei Zheng, Yujia Wang, Songling Fu, Wei Wang, Chunhong Xie, Yiying Zhang, Fangqi Gong

**Affiliations:** Department of Cardiology, Children’s Hospital, Zhejiang University School of Medicine, National Clinical Research Center for Child Health, No. 3333 Binsheng Road, Hangzhou, 310051 People’s Republic of China

**Keywords:** Predictive markers, Bioinformatics

## Abstract

Although intravenous immunoglobulin (IVIG) can effectively treat Kawasaki disease (KD), 10–20% of KD patients show no beneficial clinical response. Developing reliable criteria to discriminate non-responders is important for early planning of appropriate regimens. To predict the non-responders before IVIG treatment, gene expression dataset of 110 responders and 61 non-responders was obtained from Gene Expression Omnibus. After weighted gene co-expression network analysis, we found that modules positively correlated with the non-responders were mainly associated with myeloid cell activation. Transcripts up-regulated in the non-responders, *IL1R2, GK, HK3, C5orf32, CXCL16, NAMPT* and *EMILIN2*, were proven to play key roles via interaction with other transcripts in co-expression network. The crucial transcripts may affect the clinical response to IVIG treatment in acute KD. And these transcripts may serve as biomarkers and therapeutic targets for precise diagnosis and treatment of the non-responders.

## Introduction

Kawasaki disease (KD) is an acute, self-limited, systemic vasculitis involving the coronary arteries, which mainly occurs in children under 5 years, and KD is the most common cause of acquired heart disease in developed countries^1^. Although the pathogenesis of KD remains unknown, intravenous immunoglobulin (IVIG) could effectively decrease the incidence of coronary artery lesions (CALs)^1^. However, 10–20% of the KD patients show no beneficial clinical response^2^. The incidence of CALs was higher in IVIG non-responders than that in IVIG responders^3^. Developing reliable criteria to discriminate non-responders from responders before IVIG treatment is important for early planning of appropriate regimens. Current scoring systems have limited value for predicting IVIG resistance due to regional and ethnic diversities^4–7^; moreover, those scoring systems only include demographic and laboratory parameters, but do not consider gene expression profiling. Therefore, novel biomarkers for predicting IVIG treatment outcome are required to discriminate non-responders from responders.

With the development of high-throughput research methods, bioinformatics analysis of gene expression profiling is widely used for exploring mechanism and identifying potential biomarkers. Previous studies have suggested that IL-1 pathway may be reasonable targets for IVIG non-responders by comparing the transcript abundance between IVIG non-responders and responders^8^. CXCL12 was identified as a key candidate molecule for IVIG non-responders through comparing the gene expression profiles of iPSC-ECs generated from IVIG non-responders and responders^9^. These studies mainly focused on individual molecules which were differently expressed; however, occurrence and development of disease is a systematic biological process network, in which genes act collaboratively and those genes playing important roles may not change significantly.

Weighted gene co-expression network analysis (WGCNA)^10^ is a new tool to explore the potentially correlated modules that function in the expression data. Modules that are highly relevant to clinical trait are further analyzed and crucial transcripts are identified. WGCNA has been proven to be useful in various types of diseases, such as cancer^11^, immune disease^12^, chronic disease^13^.

Although researchers have conducted numerous bioinformatics studies to predict IVIG treatment response^8,14,15^, WGCNA has rarely been used. Herein, WGCNA was performed to confirm gene modules related to the non-responders. After we systematically analyzed the modules related to the non-responders by series of bioinformatics methods, several crucial transcripts up-regulated in the non-responders were identified and verified.

## Methods

### Data information

Gene expression dataset of GSE63881 was obtained from NCBI Gene Expression Omnibus (GEO) (https://www.ncbi.nlm.nih.gov/geo/). GSE63881 consists of expression profiles of 171 samples of blood RNA collected during the acute phase, prior to IVIG administration. The microarray platform is Illumina Human HT-12 V4.0 expression beadchip. Patients diagnosed with KD had fever for at least 3 days but not longer than 10 days, and met at least four of five clinical criteria for KD (rash, conjunctival injection, cervical lymphadenopathy, oral mucosal changes, and changes in the extremities) or three of five criteria and coronary artery abnormalities documented by echocardiogram^16^. Here we used the samples of 171 acute KD patients which contain 110 responders and 61 non-responders, and the written parental informed consent was obtained. Non-responders were defined as persistent or recrudescent fever at least 36 h after the end of their initial treatment with IVIG infusion^16^.

### Data preprocessing

Probe annotate was used to conduct expression matrix and match the probes with the gene symbols. Probes matching with multiple gene symbols were removed, and the largest values of probes were regarded as the expression values for gene symbols corresponding to multiple probes. Since transcripts with low-intensity signals are usually regarded as background noise, we chose the transcripts with intensity of more than 200 (10,621 transcripts) for subsequent analysis.

### Construction of weighted co-expression network

WGCNA R package that was originally described by Horvath and Zhang^17^ (v1.68) was used to construct gene co-expression networks with the 6000 transcripts with top variance. Firstly, the Pearson's correlation matrices were calculated for all gene pairs. Secondly, the Pearson's correlation matrices were transformed into an adjacent matrice with an appropriate soft-thresholding value β. The soft-thresholding value β was determined by the function “sft$powerEstimate”, and β = 20 was deemed as the most appropriate one when the scale-free fit index was up to 0.9. Then, converting the adjacency matrix into a topological overlap matrix (TOM) was carried out so that the indirect correlations between transcripts were concerned. Finally, Hierarchical clustering function was used to construct co-expression modules based on the TOM matrix with a minimum size of 30 transcripts. Pearson’s correlations of module eigengenes were calculated, and modules with similar eigengenes (Pearson’s correlation higher than 0.75) were merged into one module.

An adjacency heatmap with randomly selected 500 transcripts was visualized to verify the reliability of the division of modules. Besides, cluster analysis of module eigengenes was also plotted to reveal the interactions among modules.

### Identification of clinical related modules

We calculated the correlations between IVIG treatment response and modules. Modules that were positively correlated with the non-responders were considered to play an important role in IVIG non-responders. On the other hand, modules that were positively correlated with the responders were considered to play an important role during IVIG response.

Gene significance (GS) was used to represent the correlation of a transcript with the clinical trait and module membership (MM) was used to represent the correlation of a transcript with the related modules.

### Gene Ontology (GO) analysis of clinical related modules

To explore the function of clinical related modules, GO analysis was utilized to identify the biological process (BP) of the clinical related modules using the clusterProfiler R package (v3.12.0). GO terms with p-value < 0.01 were considered to be significant.

### Differentially expressed genes (DEGs) analysis

To investigate the difference of the expression profiles between non-responders and responders of transcripts within the clinical related modules, DEGs analysis was used based on Empirical Bayes test using limma R package (v3.40.6). Threshold of DEGs was set as |log2 fold-change (logFC)| > 1 and p < 0.01.

### Validation of DEGs

To verify the DEGs between non-responders and responders, we searched the DEGs in another dataset (GSE18606) and then analyzed the logFC values between non-responders and responders using limma R package (v3.40.6). Expression profiles of 20 samples of acute KD patients which contain 12 responders and 8 non-responders were obtained. Threshold of DEGs was set as |logFC| > 0.01 and p < 0.05.

### Sub-network of WGCNA

To identify crucial transcripts, the co-expression network of DEGs with GS > 0.6 and MM > 0.6 and their related transcripts were conducted using cytoscape software (v3.6.1). Transcripts with the highest intramodular connectivity in the brown module were identified as crucial transcripts and the crucial transcripts were mapped to the co-expression network.

## Results

### Data preprocessing

**Figure 1:**
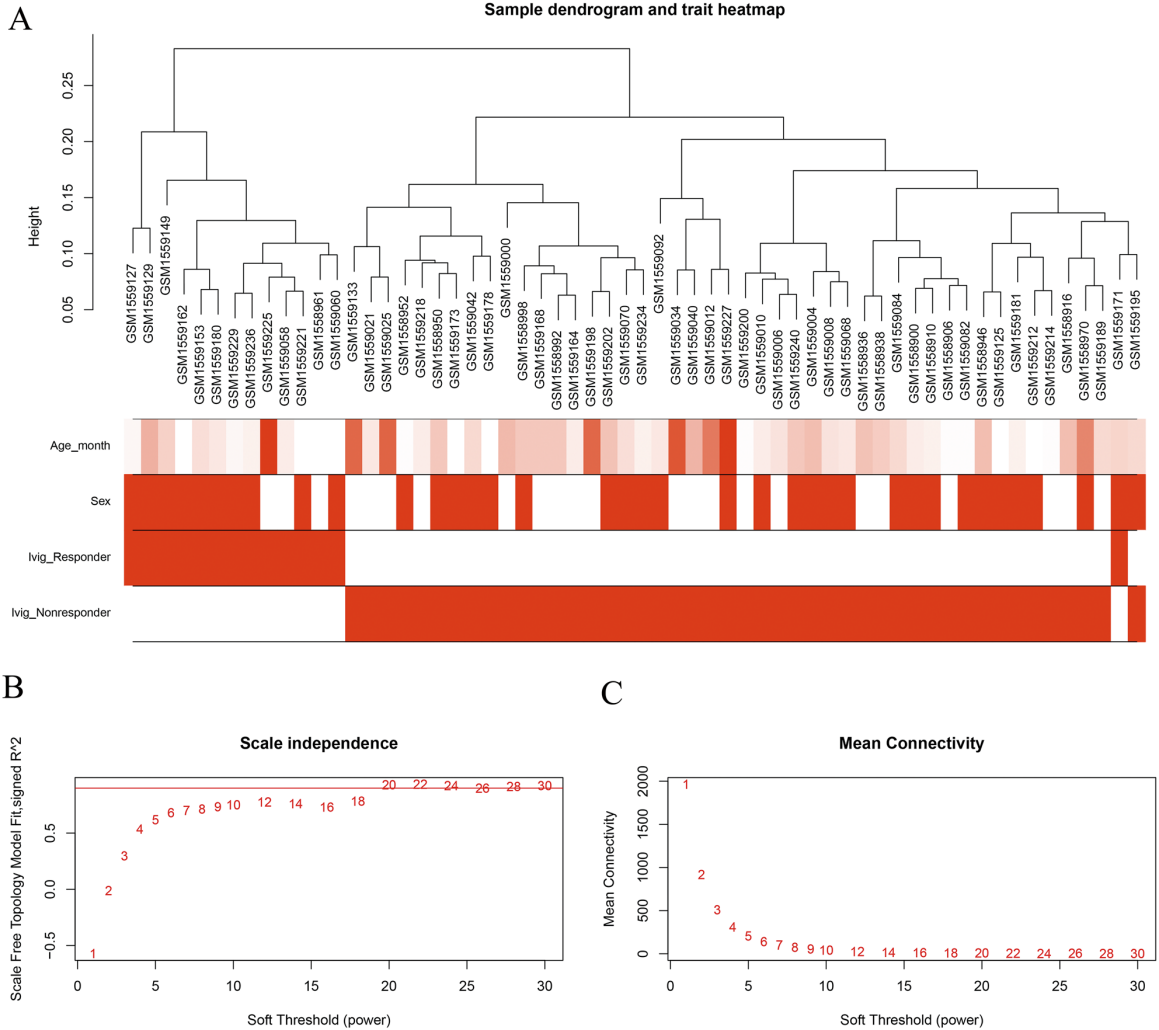
Clustering of samples and determination of soft-thresholding power. (**A**) Clustering based on the expression data of acute KD patients. Color intensity was proportional to responsive status, sex and age. (**B**) Analysis of the scale-free fit index for different soft-thresholding powers (β) ranging from 1 to 30. (**C**) Analysis of the mean connectivity for different soft-thresholding powers. β = 20 is deemed as the most appropriate one.

Gene expression dataset of GSE63881 which containing 110 responders and 61 non-responders was obtained from GEO. Clinical characteristics, including sex, age and status of coronary artery, were summarized [Supplementary Table S1 (online)]. IVIG non-responders are more prone to aneurysms, which is consistent with previous research^3^. After data preprocessing, 10,621 transcripts were obtained for subsequent analysis. Variance was calculated for these transcripts and the top 6000 transcripts with the highest variance were used to perform sample clustering analysis. A total of 46 non-responder samples and 14 responder samples were used after the outlier samples were excluded. All the samples were divided into two clusters on the whole, revealing stability within the groups and difference between groups (Fig. 1A).

### Construction of weighted co-expression network

**Figure 2:**
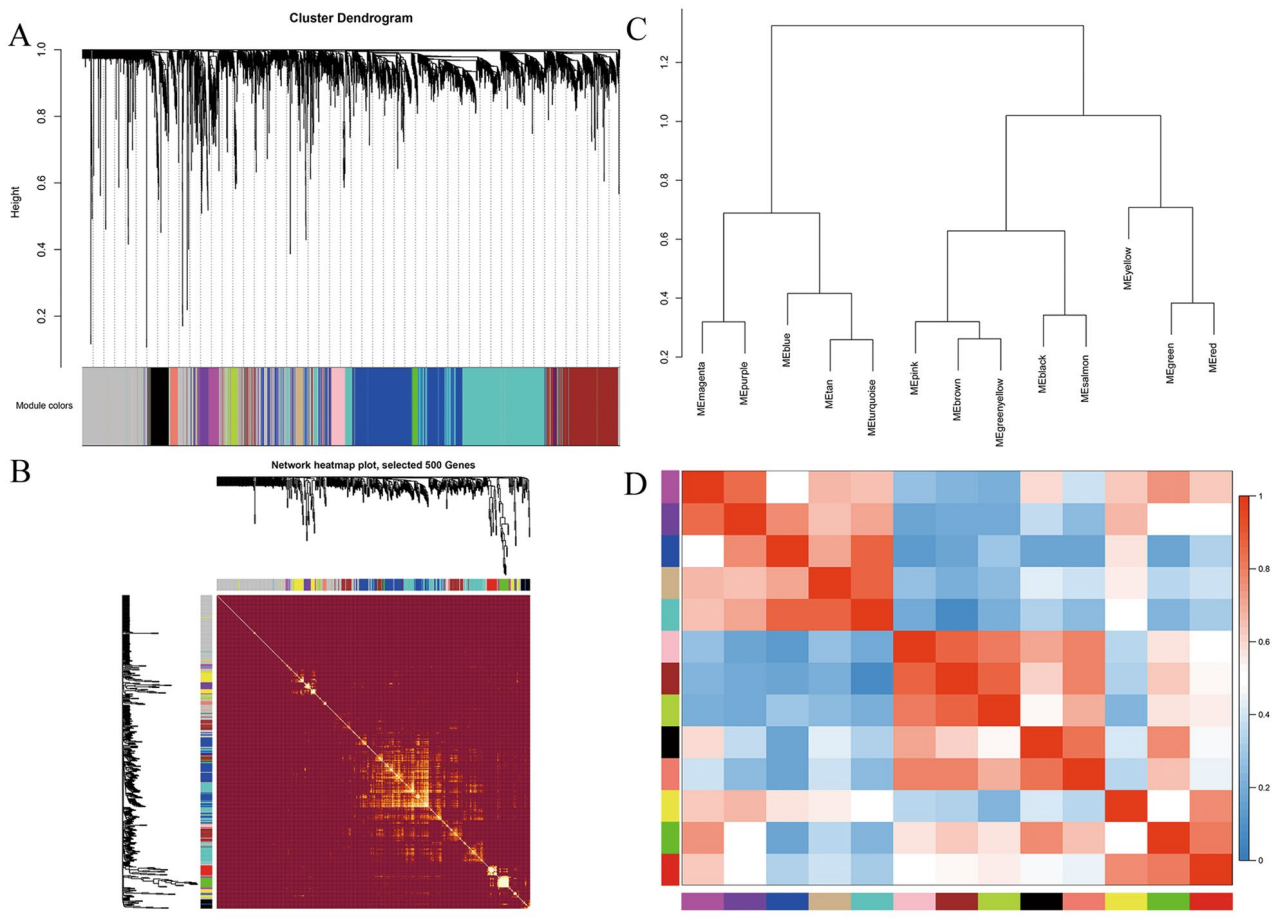
Construction of co-expression modules by WGCNA. (**A**) Custer dendrogram of genes based on module eigengenes. The colored bars below the dendrogram represent 13 different modules. (**B**) Adjacency heatmap with randomly selected 500 genes. The horizontal axis and vertical axis represent different genes within modules. The brightness of yellow represents correlation between paired genes of different modules. (**C**) Clustering dendrogram of 13 module eigengenes. (**D**) Adjacency heatmap of module eigengenes. Red represents high correlation and blue represents low correlation.

The most appropriate β was 20 and the relatively balanced scale independence and mean connectivity of the WGCNA were identified (Fig. 1B,C). After hierarchical clustering and module merging, the 6000 transcripts were divided into 13 modules (Fig. 2A) except the grey module which included the transcripts that cannot be grouped into any modules. The 13 modules include black, blue, brown, green, green yellow, magenta, pink, purple, red, salmon, tan, turquoise and yellow, containing 171, 878, 701, 222, 82, 114, 133, 85, 211, 66, 73, 1043 and 396 transcripts respectively. Figure 2B shows the correlation of transcripts within the modules. There were no significant interactions among genes within different modules, indicating the reliability of the division of modules. The clustering dendrogram indicates that the 13 modules were mainly divided into two classes, representing two main functions (Fig. 2C). The correlations between different modules are shown in Fig. 2D, and there was no significant correlation between different modules.

### Identification of clinical related modules

**Figure 3:**
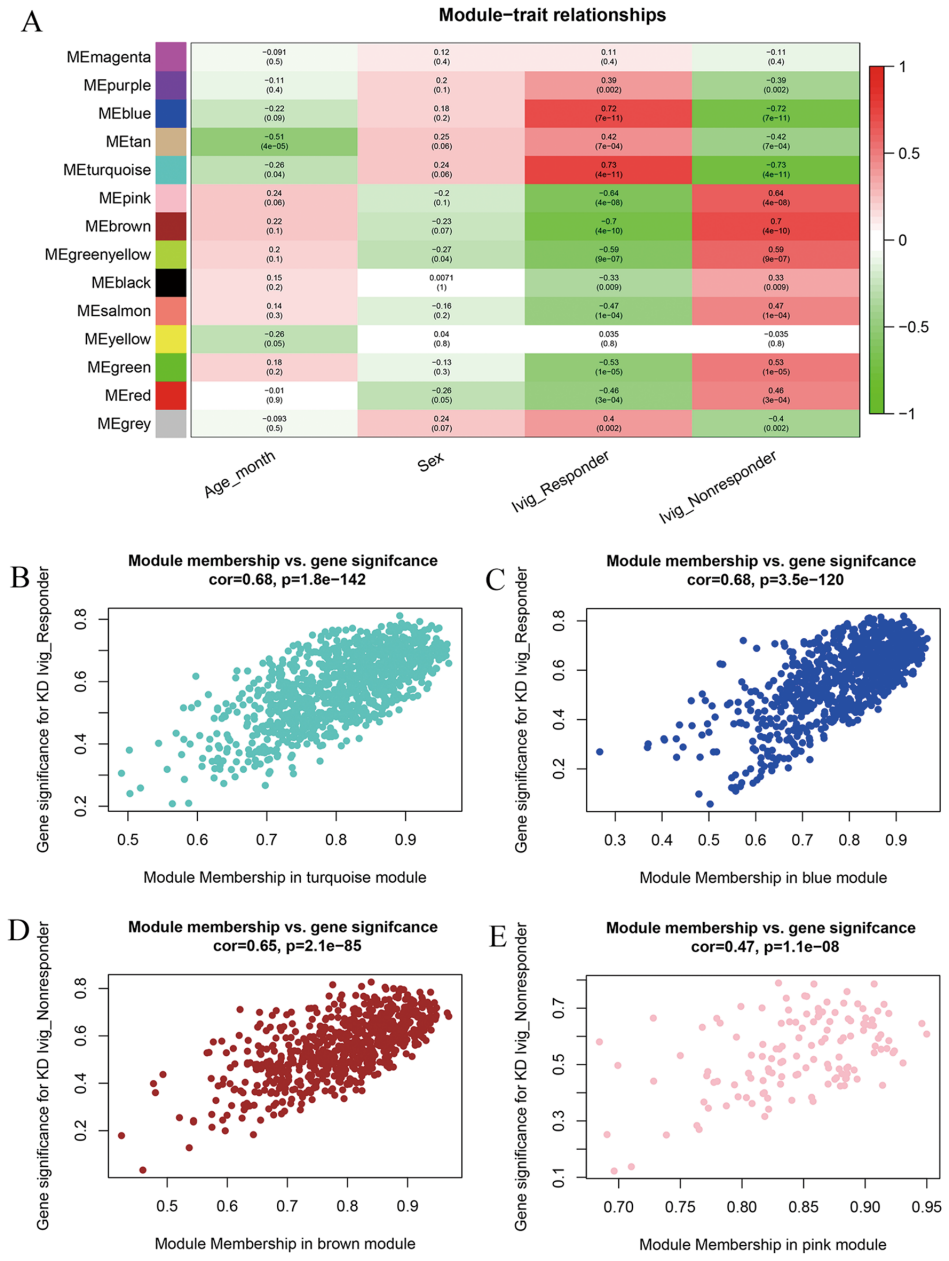
Identification of clinical related modules. (**A**) Heatmap of module-trait correlation. Number in each cell depicts the corresponding correlation coefficients and p-value. Red and green cells represent high and low correlation coefficients respectively. The blue, turquoise, pink and brown module were identified as clinical related modules. (**B**–**E**) Scatter plot for correlation between the Gene significance (GS) and Module Membership (MM) of turquoise (**B**), module (**C**), module (**D**) and pink module (**E**).

After the correlation between the interesting traits and modules was calculated, blue module and turquoise module were positively correlated with the IVIG responders, which means these two modules play an important role in IVIG response. Brown module and pink module were positively correlated with the non-responders, which were considered to play an important role in IVIG nonresponse (Fig. 3A).

Correlation of transcripts with the clinical trait and the clinical related modules were depicted in the scatter plots respectively (Fig. 3B–E). Correlation of transcripts with the clinical trait was defined as GS value and correlation of transcripts with the related modules was defined as MM value. The GS and MM values of transcripts within the blue, turquoise and brown module were quite high while that of the pink module were low.

### GO analysis of clinical related modules

**Figure 4:**
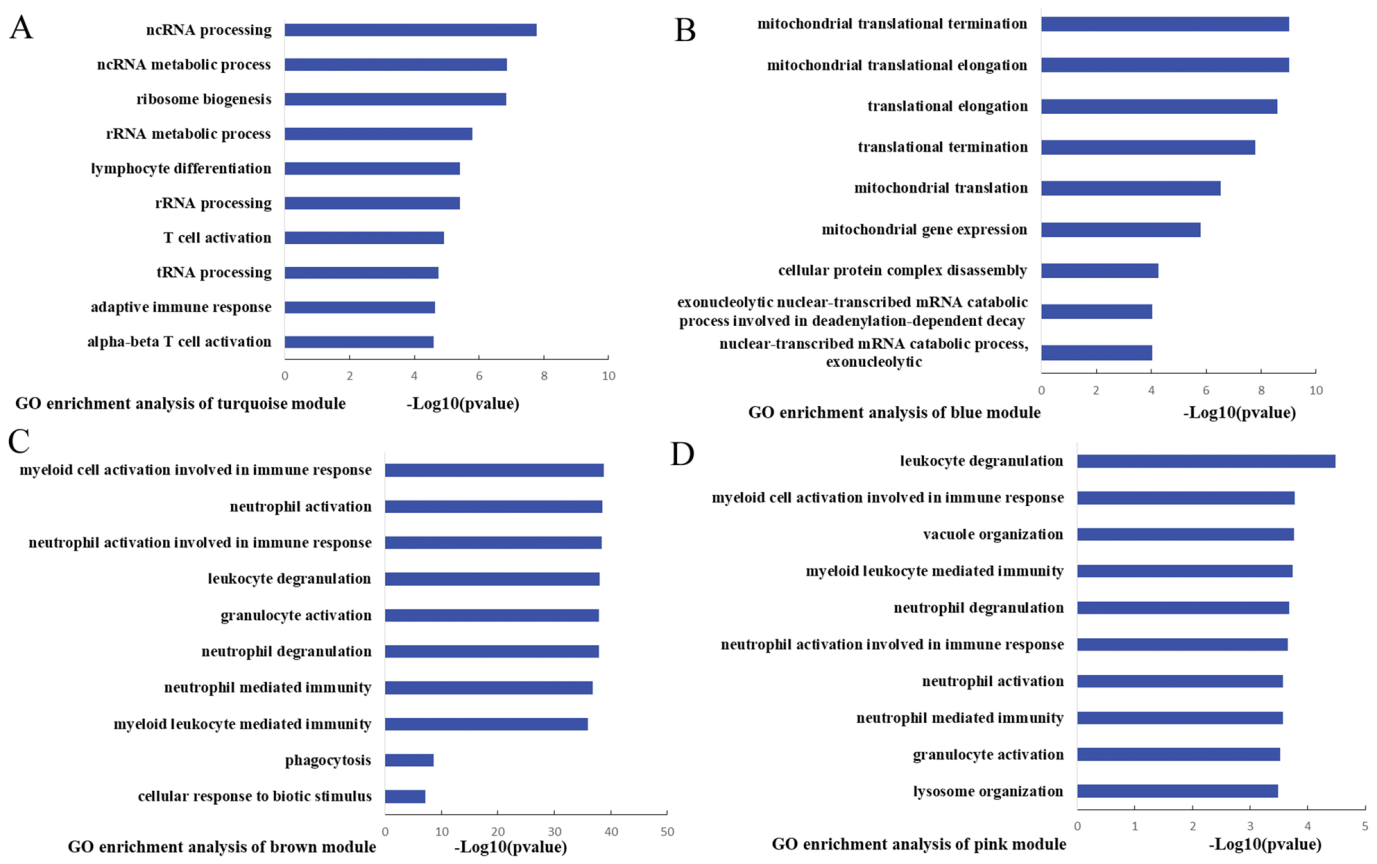
GO enrichment analyses of clinical related modules. (**A**–**D**) Top 10 significantly enriched GO-BP terms of clinical related modules. (**A**) Turquoise module (**B**) blue module (**C**) brown module (**D**) pink module.

GO analysis was utilized to explore the function of the clinical related modules. Enriched GO-BP terms that the turquoise module associated with were mainly about ncRNA, rRNA, tRNA processing and adaptive immune response, such as “ncRNA processing”, “T cell activation” and “alpha–beta T cell activation” (Fig. 4A). Enriched GO-BP terms that the blue modules which were mainly associated with mitochondrial translation, such as “mitochondrial translational termination”, “mitochondrial translational elongation” and “mitochondrial gene expression” (Fig. 4B). Enriched GO-BP terms that the brown and pink modules associated with were mainly about the immune response of myeloid cell, such as “myeloid cell activation involved in immune response”, “neutrophil activation”, “neutrophil degranulation” and “phagocytosis” (Fig. 4C,D).

### DEGs analysis

To identify the crucial transcripts that function in the non-responders, DEGs with GS > 0.5 and MM > 0.5 in the brown and pink modules were analyzed. There were 83 transcripts in the brown module up-regulated in the non-responders [Supplementary Table S2 (online)]. Additionally, 23 transcripts in pink module were up-regulated in the non-responders compared with the responders [Supplementary Table S3 (online)].

### Verification of DEGs in another dataset

**Figure 5:**
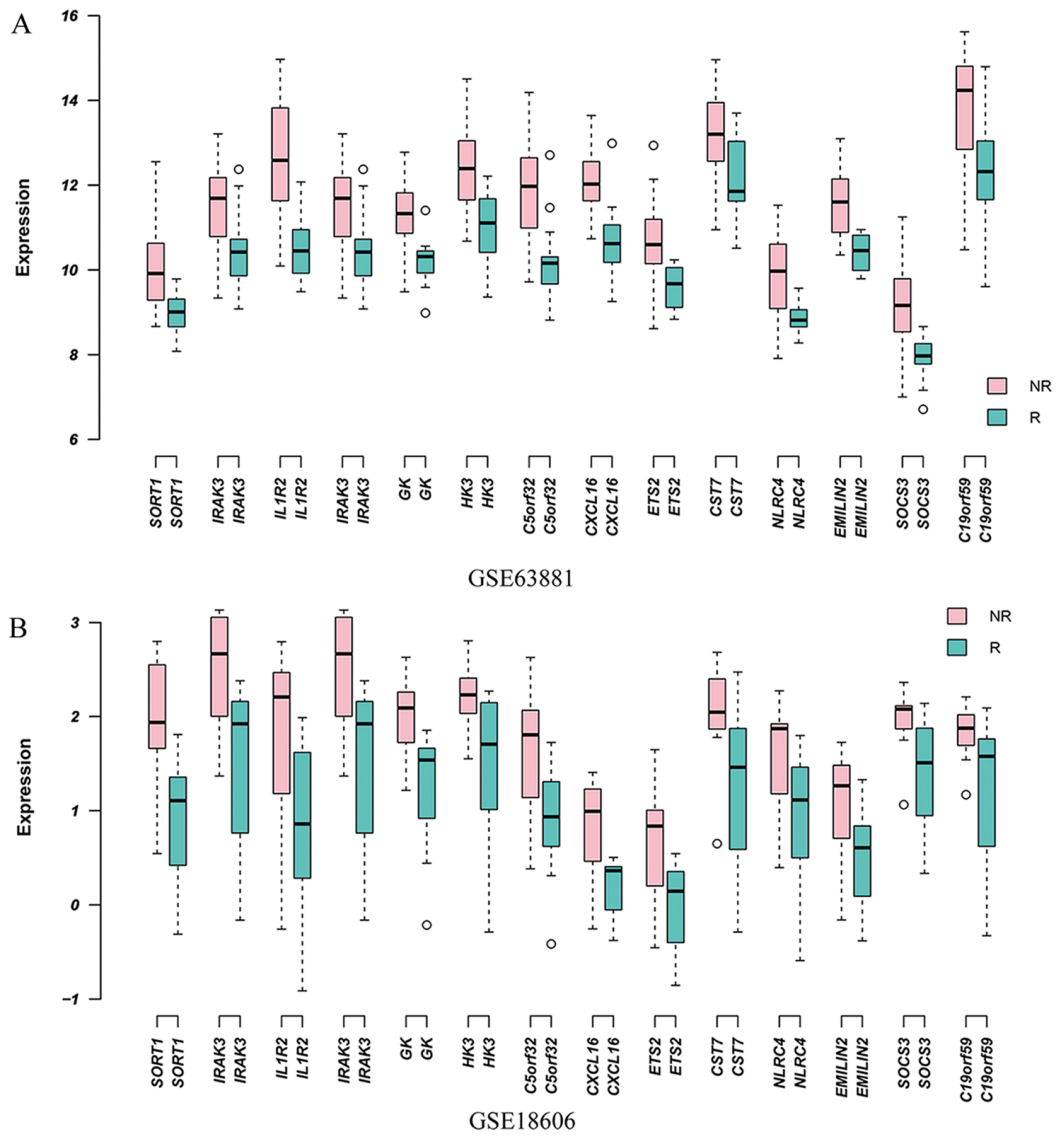
Expression levels of the DEGs in the datasets of GSE63881 and GSE18606. (**A**) Expression levels of the genes in GSE63881 (p < 0.01, logFC > 1). (**B**) Expression levels of genes in GSE18606 (p < 0.05, logFC > 1).

To verify the DEGs between non-responders and responders, the DEGs were analyzed in another dataset (GSE18606). After analyzing the DEGs in the dataset GSE18606, there were 14 transcripts up-regulated in the non-responders which were concordant with the dataset GSE63881. However, the 23 DEGs in the pink module were not differentially expressed in the dataset GSE18606. The expression levels of the DEGs in the datasets of GSE63881 and GSE18606 are shown in Fig. 5A,B.

### Identification of crucial transcripts

**Figure 6:**
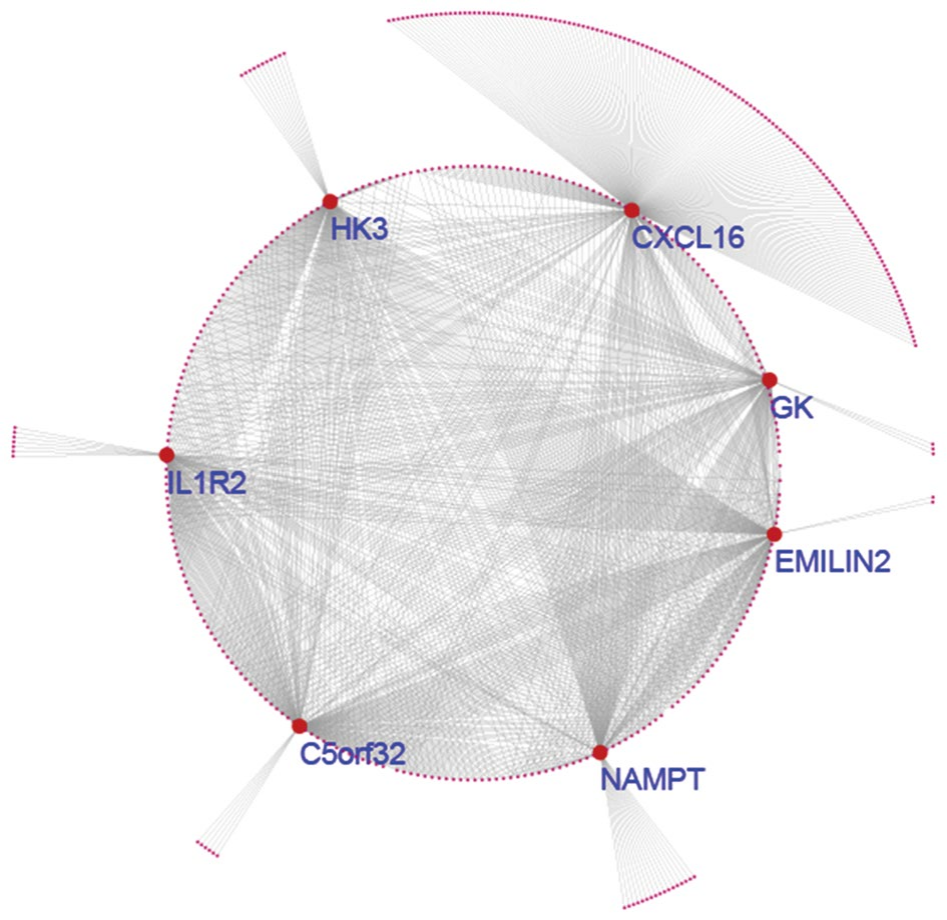
Sub-network of WGCNA based on the brown module. Red nodes represent genes and edges represent weighted correlation. The crucial genes are clearly showed.

To identify the crucial transcripts, the verified DEGs with GS > 0.6 and MM > 0.6 were conducted to construct a co-expression network. The GS and MM values of the DEGs are listed in Supplementary Table S4 (online). The transcripts, *IL1R2, GK, HK3, C5orf32, CXCL16, NAMPT* and *EMILIN2,* were proven to be crucial transcripts that may play crucial roles in IVIG non-responders (Fig. 6).

## Discussion

There are still no reliable biomarkers to discriminate non-responders from responders before IVIG treatment in acute KD. It is imperative to reveal the underlying molecular mechanisms and pathological processes governing KD and IVIG therapy. High-throughput research methods revealed that IVIG nonresponse is associated with SNP mutations^18,19^, DNA methylation^15^, lncRNA^14^ and miRNA^20^. As for transcripts, IL-1 pathway genes^8^, ankyrinD22, carcinoembryonic antigen cell adhesion molecule 1 (*CEACAM1*), fructose-2, 6 biphosphatase 2 (*PFKB2*), haptoglobin (*HP*) and matrix metalloproteinase-8 (*MMP-8*)^16^ may be reasonable for IVIG nonresponse. However, researches mainly focused on studying individual molecules, and there is still no consistent conclusions on the mechanism of IVIG nonresponse. As occurrence and development of disease is a complex biological network, constructing gene regulatory networks and uncovering the crucial transcripts is necessary. Presently, we used the gene expression dataset of GSE63881 and GSE18606 to screen the crucial transcripts involved in IVIG nonresponse by WGCNA.

WGCNA was performed and 6000 genes with the top variance were divided into 13 modules including four clinical related modules. The blue module and the turquoise module were highly correlated with the responders, while the brown module and the pink module were highly correlated with the non-responders which were worthy of subsequent analysis. To elucidate the function of these clinical related modules, GO enrichment analysis was performed. The turquoise module was mainly about ncRNA, rRNA, tRNA processing and adaptive immune response while the blue module was mainly about mitochondrial translation. Several studies have identified that ncRNA, such as miRNA^21^ and lncRNA^14^, paly a variety of roles in the occurrence of KD. As for mitochondrial translation, previous studies found two different populations of platelets with different mitochondrial functions in KD patients which may affect the inflammatory responses^22^. These results imply that IVIG may function through the regulation of ncRNA, mitochondrial translation and adaptive immune response to treat KD patients. The brown module and the pink module correlated with IVIG non-responders were mainly about the immune response of myeloid cell, such as “neutrophil activation”, “neutrophil degranulation”, and “phagocytosis”. Studies have demonstrated that neutrophil-to-lymphocyte ratio may be used as a biomarker for detecting IVIG-resistant KD, especially after the initial treatment of IVIG, implying the key functions of neutrophils^23^. Moreover, neutrophils were involved in the pathogenesis of KD^14,24^, and suppressing neutrophil activation prove effective^25^. Neutrophils have a restricted set of pro-inflammatory functions^26^, suggesting that neutrophil/myeloid activation may cause endothelial cells damage in KD.

The transcripts verified to be up-regulated in non-responders compared with responders, *IL1R2, CXCL16, C5orf32, GK, HK3, NAMPT* and *EMILIN2,* may play crucial roles in IVIG non-responders. IL1R2 is one of the negative regulators of the IL-1 system and it binds IL-1α and IL-1β with high affinity but does not induce signaling^27^. Recently, it has been shown that *Staphylococcus aureus* induces IL-1R2 shedding and consequently reducing IL-1β availability, therefore negatively modulating the subsequent inflammatory response and contributing to the bacterial persistence in blood^28^. Consistent with previous studies^8^, our study showed that *IL1R2* was up-regulated in non-responders. IL1R2 may represent a novel mechanism of IVIG nonresponse through regulation of IL-1 pathway. CXCL16 is a membrane-bound chemokine expressed in various cells, such as macrophages^29^, dendritic cells^30^ and aortic smooth muscle cells^31^, and it induces the migration of neutrophils and monocytes through its receptor named CXC chemokine receptor 6 (CXCR6). Recently, increasing evidence has indicated that CXCL16 is involved in inflammatory disease, such as acute coronary syndromes^32^ and psoriasis^33^. Therefore, we infer that the up-regulated CXCL16 may function with CXCR6 to regulate IVIG nonresponse. *C5orf32* is also known as cysteine rich transmembrane module containing 1 (*CYSTM1*), may be associated with resistance to deleterious substances^34^ and Huntington’s disease^35^. The proteins encoded by *GK* and *HK3* are involved in glucose metabolism pathways. Nicotinamide phosphoribosyl transferase, the protein encoded by *NAMPT,* is associated with oxidative stress response, apoptosis, lipid and glucose metabolism, inflammation, insulin resistance^36^ and vascular repair^37^. EMILIN2, mlastin microfibril interface located protein 2, regulates platelet activation, thrombus formation, and clot retraction^38^ and play important roles in the tumor microenvironment through affecting angiogenesis and lymphangiogenesis^39^. As for the above transcripts, very little research has been done on KD and they are worthy of further studies to assess the underlying molecular mechanisms of IVIG resistance.

There are several limitations to our study. To confirm the accuracy of the results, more patient samples and multiple methods should be used to study the results. These transcripts are from the whole blood cells and further studies are needed to identify which kind of blood cells playing a key role in the pathological process of IVIG nonresponse.

In conclusion, myeloid cell activation was identified to be associated with IVIG non-responders. The crucial transcripts, *IL1R2, GK, HK3, C5orf32, CXCL16, NAMPT* and *EMILIN2*, may affect the clinical response before initial immunoglobulin treatment in acute KD. Moreover, these crucial transcripts may serve as biomarkers and therapeutic targets for non-responders in the future.

## Supplementary information

Supplementary Tables.

## Data Availability

The datasets analyzed during the current study are available from the corresponding author on reasonable request.
