# Supplementary material for: Crucial transcripts predict response to initial immunoglobulin treatment in acute Kawasaki disease
Source: Sci Rep. 2020 Oct 20;10:17860. doi: 10.1038/s41598-020-75039-z (PMC7575539; doi:10.1038/s41598-020-75039-z)
Supplement: Supplementary file 1 — Supplementary Tables. [file 41598_2020_75039_MOESM1_ESM.docx]

**Crucial Transcripts Predict Response to Initial Immunoglobulin Treatment in Acute Kawasaki Disease**

Zhimin Geng ^1#^, Jingjing Liu ^1#^, Jian Hu^1^, Ying Wang^1^, Yijing Tao^1^, Fenglei Zheng^1^, Yujia Wang^1^, Songling Fu^1^, Wei Wang^1^, Chunhong Xie^1^, Yiying Zhang^1^, Fangqi Gong^1^*

^#^ These two authors contributed equally to this work.

**^1^** Department of Cardiology, Children's Hospital, Zhejiang University School of Medicine, National Clinical Research Center for Child Health. No. 3333 Binsheng Road, Hangzhou, 310051, PR China.

*** Corresponding author:** Fangqi Gong, Department of Cardiology, The Children’s Hospital of Zhejiang University School of Medicine, National Clinical Research Center for Child Health. No.3333, Binsheng Road, Hangzhou 310052, PR China. Tel/Fax: +86-571-886670008, E-mail: [gongfangqi@zju.edu.cn](mailto:gongfangqi@zju.edu.cn)

Table S1. Clinical characteristics of IVIG-responder and non-responder subjects.

|  | **Responder**  **(n = 110)** | | | **Non-responder**  **(n = 61)** | ***P* value^a^** |
| --- | --- | --- | --- | --- | --- |
| **Sex,** **n (%)** |  | | |  |  |
| Male | 65(59) | | 37(61) | | > 0.05 |
| Female | 45(41) | | | 24(39) |  |
| **Age (months), n (%)** | |  | |  |  |
| < 12 months | 18(16) | | | 13(21) | > 0.05 |
| 12 - 60 months | 72(66) | | | 41(67) |  |
| > 60 months | 20(18) | | | 7(12) |  |
| **CA^b^ status, n (%)** |  | | |  |  |
| Normal | 81(74) | | | 34(56) | < 0.01 |
| Dilated | 24(22) | | | 12(20) |  |
| Aneurysm | 5(4) | | | 15(24) |  |

^a^ *P* values were calculated by Chi-square test.

^b^ CA, Coronary artery.

**Table S2.** Transcripts up-regulated in the non-responders in the brown module

| **Gene Symbol** | **logFC(NR/R)** | **P.Value** | **adj.P.Value** |
| --- | --- | --- | --- |
| IL1R2 | 2.164988169 | 4.48E-07 | 3.64E-06 |
| HP | 1.933805552 | 2.45E-05 | 9.47E-05 |
| DYSF | 1.901288178 | 2.04E-12 | 2.80E-09 |
| LRG1 | 1.767809628 | 7.48E-09 | 1.94E-07 |
| MMP9 | 1.646521857 | 1.53E-05 | 6.41E-05 |
| CA4 | 1.620498299 | 2.48E-07 | 2.30E-06 |
| C5orf32 | 1.610569557 | 2.06E-06 | 1.23E-05 |
| ALPL | 1.597957869 | 1.72E-09 | 6.91E-08 |
| PGD | 1.531189245 | 3.67E-09 | 1.18E-07 |
| CXCR1 | 1.462184417 | 4.63E-10 | 2.90E-08 |
| C19orf59 | 1.425825658 | 0.0003606 | 0.000962591 |
| CXCL16 | 1.381788263 | 3.93E-08 | 5.88E-07 |
| LOC100130914 | 1.367473275 | 7.66E-10 | 4.09E-08 |
| HK3 | 1.363218656 | 4.66E-06 | 2.39E-05 |
| LOC440731 | 1.362378933 | 4.38E-07 | 3.57E-06 |
| FAM129A | 1.357347064 | 1.33E-12 | 2.80E-09 |
| LOC642103 | 1.328238336 | 1.58E-06 | 9.94E-06 |
| ROPN1L | 1.325673886 | 1.29E-08 | 2.81E-07 |
| LOC100128326 | 1.30305636 | 7.64E-09 | 1.96E-07 |
| C1orf24 | 1.297241707 | 4.21E-10 | 2.74E-08 |
| LOC729660 | 1.285723727 | 1.79E-08 | 3.51E-07 |
| SOCS3 | 1.260283141 | 1.94E-06 | 1.17E-05 |
| LOC441124 | 1.258238233 | 1.87E-08 | 3.62E-07 |
| MGAM | 1.253628275 | 1.62E-06 | 1.01E-05 |
| LOC401357 | 1.225141365 | 3.92E-08 | 5.87E-07 |
| STXBP2 | 1.221109511 | 3.26E-06 | 1.79E-05 |
| PTAFR | 1.218435318 | 2.49E-08 | 4.28E-07 |
| FPR1 | 1.197857553 | 1.35E-09 | 5.95E-08 |
| FCGR2A | 1.189669991 | 9.28E-09 | 2.26E-07 |
| CEACAM1 | 1.188602674 | 0.0009223 | 0.002185136 |
| SERPINA1 | 1.180454795 | 8.23E-09 | 2.07E-07 |
| FCGR1C | 1.180443709 | 0.0001239 | 0.000378597 |
| S100A11 | 1.177642768 | 1.14E-08 | 2.56E-07 |
| MMP25 | 1.174816085 | 9.73E-07 | 6.67E-06 |
| CDA | 1.173211465 | 6.87E-07 | 5.07E-06 |
| LOC100170939 | 1.149701192 | 0.0001558 | 0.000461844 |
| SIPA1L2 | 1.143292991 | 1.34E-05 | 5.76E-05 |
| CMTM2 | 1.142542194 | 0.0001142 | 0.000353927 |
| LILRA6 | 1.139286728 | 9.33E-06 | 4.25E-05 |
| GAS7 | 1.134843994 | 9.22E-07 | 6.38E-06 |
| PYGL | 1.133003757 | 2.38E-08 | 4.16E-07 |
| HIST2H2AA4 | 1.132416755 | 1.61E-05 | 6.67E-05 |
| SIGLEC10 | 1.129951883 | 3.55E-05 | 0.000129565 |
| EMILIN2 | 1.129019971 | 4.04E-07 | 3.33E-06 |
| LOC153561 | 1.121903333 | 0.000192 | 0.000552768 |
| IMPA2 | 1.114667201 | 8.03E-07 | 5.71E-06 |
| SLC11A1 | 1.114452855 | 6.98E-07 | 5.12E-06 |
| FLOT1 | 1.113712966 | 8.22E-07 | 5.80E-06 |
| HCK | 1.109018044 | 3.27E-08 | 5.10E-07 |
| NAMPT | 1.10162113 | 9.29E-06 | 4.24E-05 |
| SLC22A4 | 1.098342093 | 1.15E-05 | 5.06E-05 |
| GK | 1.09188424 | 9.80E-07 | 6.70E-06 |
| PFKFB3 | 1.090034956 | 0.0001309 | 0.000397223 |
| NCF4 | 1.08768323 | 3.42E-08 | 5.30E-07 |
| LOC648984 | 1.086756741 | 0.0007185 | 0.00175544 |
| POR | 1.085963653 | 2.23E-07 | 2.13E-06 |
| CST7 | 1.085813416 | 0.0001544 | 0.000458267 |
| EMR2 | 1.082409766 | 7.81E-07 | 5.61E-06 |
| FCGR1A | 1.076107451 | 0.0009345 | 0.002209745 |
| NLRC4 | 1.075920594 | 3.30E-05 | 0.000122447 |
| RNF24 | 1.073855658 | 2.24E-07 | 2.13E-06 |
| ETS2 | 1.068404978 | 2.99E-05 | 0.000112697 |
| UPP1 | 1.06696348 | 2.33E-05 | 9.08E-05 |
| SORL1 | 1.066216454 | 8.66E-07 | 6.04E-06 |
| SLC25A37 | 1.062746147 | 4.63E-13 | 2.80E-09 |
| SORT1 | 1.060752715 | 3.88E-05 | 0.000140095 |
| RFX2 | 1.059261732 | 1.90E-08 | 3.65E-07 |
| ITGAM | 1.058047304 | 2.73E-06 | 1.55E-05 |
| ACSL1 | 1.057354181 | 3.79E-06 | 2.01E-05 |
| AGTRAP | 1.046642992 | 8.13E-08 | 9.89E-07 |
| PDLIM7 | 1.045281487 | 1.32E-06 | 8.58E-06 |
| MANSC1 | 1.032842419 | 1.66E-06 | 1.04E-05 |
| ST3GAL4 | 1.029626277 | 4.72E-06 | 2.41E-05 |
| NCF2 | 1.029552048 | 1.20E-07 | 1.34E-06 |
| TLR5 | 1.023750027 | 0.0001246 | 0.000380223 |
| C5AR1 | 1.021688183 | 3.87E-07 | 3.23E-06 |
| CKAP4 | 1.01875705 | 0.000262 | 0.000725288 |
| DUSP1 | 1.012191209 | 5.78E-07 | 4.42E-06 |
| IRAK3 | 1.006764729 | 0.0009037 | 0.00214563 |
| PVRL2 | 1.006260344 | 3.73E-08 | 5.64E-07 |
| SLC2A14 | 1.003351506 | 8.01E-05 | 0.000259599 |
| NLRP12 | 1.002065371 | 5.53E-07 | 4.28E-06 |
| FPR2 | 1.001562675 | 3.35E-06 | 1.83E-05 |

**Table S3.** Transcripts up-regulated in the non-responders in the pink module

| **Gene Symbol** | **logFC(NR/R)** | **P.Value** | **adj.P.Value** |
| --- | --- | --- | --- |
| LOC100134634 | 1.601703046 | 3.88E-12 | 3.38E-09 |
| LOC729021 | 1.570956274 | 4.63E-12 | 3.38E-09 |
| LOC100134530 | 1.622879497 | 2.69E-11 | 5.99E-09 |
| MBOAT7 | 1.529327932 | 1.04E-10 | 1.21E-08 |
| LOC730284 | 1.492608076 | 2.78E-10 | 2.07E-08 |
| LOC100132491 | 1.41899703 | 3.86E-10 | 2.59E-08 |
| LOC100132112 | 1.385645232 | 3.90E-10 | 2.59E-08 |
| LOC730286 | 1.644931284 | 4.72E-10 | 2.94E-08 |
| LOC100134734 | 1.14296561 | 4.38E-09 | 1.34E-07 |
| CTSA | 1.210437115 | 6.44E-09 | 1.77E-07 |
| LOC100132960 | 1.018968189 | 1.01E-08 | 2.40E-07 |
| LOC728728 | 1.297338453 | 1.05E-08 | 2.45E-07 |
| LOC399744 | 1.065015743 | 1.24E-08 | 2.71E-07 |
| LOC728440 | 1.057586575 | 1.31E-08 | 2.83E-07 |
| PHC2 | 1.279247624 | 4.07E-08 | 6.03E-07 |
| ANPEP | 1.453193004 | 4.81E-08 | 6.75E-07 |
| ATP6V0D1 | 1.045504013 | 9.92E-08 | 1.16E-06 |
| LOC646301 | 1.279117366 | 1.03E-07 | 1.20E-06 |
| SEMA4A | 1.168019023 | 1.83E-07 | 1.83E-06 |
| IL8RB | 1.412564489 | 2.43E-07 | 2.27E-06 |
| VASP | 1.018685665 | 1.10E-06 | 7.32E-06 |
| CTSD | 1.039876836 | 7.62E-06 | 3.60E-05 |
| GRN | 1.140371839 | 1.39E-05 | 5.92E-05 |

**Table S4.** Differentially expressed genes with GS > 0.6 and MM > 0.6

| **Gene Symbol** | **GS** | **p.GS** | **MMbrown** | **p.MM.brown** |
| --- | --- | --- | --- | --- |
| CXCL16 | 0.704941654 | 3.24E-10 | 0.938209385 | 2.07E-28 |
| NAMPT | 0.683371758 | 1.78E-09 | 0.839400348 | 5.42E-17 |
| EMILIN2 | 0.671615419 | 4.25E-09 | 0.892212773 | 1.10E-21 |
| GK | 0.657855179 | 1.12E-08 | 0.904237627 | 4.24E-23 |
| HK3 | 0.623891691 | 1.01E-07 | 0.890532072 | 1.69E-21 |
| IL1R2 | 0.619180795 | 1.34E-07 | 0.850206194 | 8.41E-18 |
| C5orf32 | 0.604315782 | 3.18E-07 | 0.87715919 | 3.94E-20 |
| CST7 | 0.591719232 | 6.39175E-07 | 0.813083597 | 3E-15 |
| SOCS3 | 0.578808884 | 1.27029E-06 | 0.748757126 | 6E-12 |
| ETS2 | 0.56576959 | 2.47E-06 | 0.892459396 | 1.04E-21 |
| NLRC4 | 0.551030743 | 5.06E-06 | 0.860289282 | 1.29E-18 |
| C19orf59 | 0.538095293 | 9.24E-06 | 0.81654118 | 1.83E-15 |
| SORT1 | 0.531869832 | 1.22E-05 | 0.81068164 | 4.19E-15 |
| IRAK3 | 0.524276031 | 1.71E-05 | 0.821588609 | 8.80E-16 |
